# North American population-based validation of the National Comprehensive Cancer Network Practice Guideline Recommendations for locoregional lymph node and bone imaging in prostate cancer patients

**DOI:** 10.1038/s41416-018-0323-3

**Published:** 2018-11-14

**Authors:** Felix Preisser, Elio Mazzone, Sebastiano Nazzani, Michele Marchioni, Marco Bandini, Zhe Tian, Fred Saad, Denis Soulières, Shahrokh F. Shariat, Francesco Montorsi, Hartwig Huland, Markus Graefen, Derya Tilki, Pierre I. Karakiewicz

**Affiliations:** 1 Martini-Klinik Prostate Cancer Center, University Hospital Hamburg-Eppendorf, Hamburg, Germany; 2 Cancer Prognostics and Health Outcomes Unit, Division of Urology, University of Montreal Health Center, Montreal, Quebec Canada; 3 Department of Urology and Division of Experimental Oncology, URI, Urological Research Institute, IRCCS San Raffaele Scientific Institute, Milan, Italy; 4 Academic Department of Urology, IRCCS Policlinico San Donato, University of Milan, Milan, Italy; 5 Department of Urology, SS Annunziata Hospital, “G. D’Annunzio” University of Chieti, Chieti, Italy; 6 Centre Hospitalier de l’Université de Montréal, Montreal, Canada; 7 Department of Urology, Medical University of Vienna, Vienna, Austria; 8 Department of Urology, University Hospital Hamburg-Eppendorf, Hamburg, Germany

**Keywords:** Metastasis, Prostate cancer, Cancer imaging

## Abstract

**Background:**

The National Comprehensive Cancer Network (NCCN) guidelines provide recommendations for staging of prostate cancer patients in the objective regarding presence of locoregional lymph node metastases (LNM) and bone metastases. We tested the performance characteristics of these recommendations in a community setting.

**Methods:**

Within the Surveillance, Epidemiology, and End Results database (2004–2014), we identified patients with available Gleason, clinical stage and prostatic specific antigen. Performance characteristics endpoints consisted of sensitivity, specificity, positive predictive value (PPV), negative predictive value (NVP), overall accuracy and the number needed to image (NNI).

**Results:**

Totally, 191,308 patients were assessable for the validation of the LNM staging recommendations. Sensitivity ranged from 80.6 to 86.3%, specificity from 74.7 to 79.3%, PPV from 7.8 to 8.0%, overall accuracy from 75.0 to 79.3% and NPV was 99.5%. The respective NNI values were 12.5 and 12.8. 197,408 patients were assessable for the validation of bone scan recommendations. These recommendations resulted in 90.8% sensitivity, 76.3% specificity, PPV of 5.7%, NPV of 99.8% and overall accuracy of 76.5%. The NNI was 17.5.

**Conclusion:**

The NCCN recommendations for locoregional LNM miss few patients with clinical LNM (0.3–0.4%) and provide a virtually perfect NPV of 99.5%. Also, the recommendations for bone scan miss a marginal number of patients with established bone metastases (0.14%) and yield a virtually perfect NPV of 99.8%.

## Introduction

Clinical staging in the context of prostate cancer (PCa) is important.^1^ Treatment of individuals with locally advanced or metastatic PCa differs from that for individuals with localised PCa. The distinction between localised PCa vs. locoregional lymph node metastatic PCa and PCa metastatic to bone can be made using the recommendations of the National Comprehensive Cancer Network (NCCN), as outlined in the clinical practice guidelines in oncology.^2^

Specifically, for staging of patients with suspected locoregional lymph node metastases (LNM) the NCCN PCa guideline (Version 2.2017) recommends a pelvic computed tomography scan (CT) or pelvic magnetic resonance imaging (MRI) if clinical tumour stage is T3 or T4 or in clinical tumour stage T1 or T2 when nomogram derived LNM probability is >10%.^2^

Similarly, for staging of patients with suspected bone metastases, the NCCN PCa guideline recommends a bone scan in clinical tumour stage T1 patients when prostatic specific antigen value (PSA) is >20 ng/ml or if clinical tumour stage is T2 when PSA is >10 ng/ml or if clinical tumour stage is T3 or T4 or if Gleason score is ≥8 or if patients are symptomatic.^2^

However, to the best of our knowledge these recommendations have never been validated in a community setting within a large epidemiological database, such as the Surveillance, Epidemiology, and End Results (SEER) database. Within the current manuscript, we tested the performance characteristics of the NCCN guidelines regarding presence of locoregional LNM, as well as regarding presence of bone metastases. Specifically, we hypothesised that the use of the NCCN guidelines will not result in more than a marginal proportion of patients with missed locoregional LNM or missed bone metastases.

## Patients and methods

### Study population

Within the SEER database (2004–2014), we identified patients ≤90 years old with available information on Gleason grade group (GGG),^3^ clinical tumour stage and PSA value. Patients with a serum PSA value ≥98 ng/ml were excluded.

### Testing of the NCCN recommendations

We performed two separate analyses. First, we tested the NCCN guideline recommendations for identification of patients with positive clinical lymph node status (cN1).^4^ The NCCN recommends a pelvic CT/MRI if clinical tumour stage is T3 or T4 or if clinical tumour stage is T1 or T2 and the nomogram derived probability of LNM is >10%. Since two different nomograms can be applied and no specific recommendation is made for the use of one vs. the other, we performed two separate analyses.^5,6^ The first analysis relied on the use of the updated Briganti et al.^5^ nomogram that includes biopsy core information. The second analysis relied on the use of the online Memorial Sloan Kettering Cancer Center (MSKCC) dynamic prostate cancer nomogram that includes biopsy core information.^6^ Only patients with known positive clinical lymph node status (cN1) or negative clinical lymph node status (cN0) were included. These selection criteria resulted in 191,308 assessable patients for testing of the NCCN recommendations.

Second, we tested the NCCN guideline recommendations for identification of patients with bone metastases. The NCCN recommends a bone scan if clinical tumour stage is T1 and PSA > 20 ng/ml or if clinical tumour stage is T2 and PSA > 10 ng/ml or if clinical tumour stage is T3 or T4 or if Gleason Score is ≥8 (GGG ≥ 4). Only patients with known bone metastases (M1b) or without metastases (M0) were included. These selection criteria resulted in 197,408 assessable patients for testing the NCCN bone scan recommendations.

### Statistical analyses

Descriptive statistics consisted of frequencies and proportions for categorical variables. Means, medians and ranges were reported for continuously coded variables. The chi-square tested the statistical significance of proportions’ differences. The *t* test and Kruskal–Wallis test examined the statistical significance of means and medians differences, respectively.

The specific performance characteristics for each of the guideline recommendations consisted of sensitivity, specificity, positive predictive value (PPV), negative predictive value (NVP), overall accuracy and the number needed to image (NNI). Finally, the positive (sensitivity/(1-specificity)) and negative likelihood ratios ((1 − sensitivity)/specificity) for each recommendation were calculated. R software environment for statistical computing and graphics (version 3.4.0) was used for all statistical analyses. All tests were two sided with a level of significance set at *p* < 0.05.

## Results

### NCCN recommendations to perform a pelvic CT or MRI

#### Study population

Of 191,308 assessable patients (Tables 1), 2.3% (*n* = 4446) had clinical lymph node metastases. Patients with lymph node metastases more frequently had a PSA value > 20 ng/ml (36.7 vs. 7.5%, *p* < 0.001), more frequently harboured clinical tumour stage T3 to T4 (24.1 vs. 2.3%, *p* < 0.001) and more frequently harboured GGG 5 (42.5 vs. 6.3%, *p* < 0.001).

**Table 1:** SEER-population with prostate cancer for the validation of the bone scan recommendations, stratified according to metastatic status and for the validation of the pelvic CT/MRI recommendations, stratified according to metastatic status and lymph node status

	Overall (*n* = 197,408)	Non-metastatic (*n* = 194,342) (98.4%)	Bone metastatic (*n* = 3,066) (1.6%)	*p* Value	Overall (*n* = 191,308)	Lymph node-negative (*n* = 186,862) (97.7%)	Lymph node-positive (*n* = 4,446) (2.3%)	*p* Value
*Age*								
Median (interquartile range)	65 (59–71)	65 (59–71)	70 (62–77)	<0.001	65 (59–71)	65 (59–71)	65 (59–70)	<0.001
*Year of diagnosis*								
Mean (STE)	2011.8 (0.003)	2011.8 (0.003)	2012.1 (0.026)	<0.001	2011.8 (0.003)	2011.8 (0.003)	2012.2 (0.021)	<0.001
*PSA*								
≤10	150,991 (76.5)	150,333 (77.4)	658 (21.3)	<0.001	146,392 (76.5)	144,669 (77.4)	1723 (38.8)	<0.001
10.1–20	30,261 (15.3)	29,615 (15.2)	646 (21.2)		29,262 (15.3)	28,173 (15.1)	1089 (24.5)	
>20	16,156 (8.2)	14,394 (7.4)	1762 (57.5)		15,654 (8.2)	14,020 (7.5)	1634 (36.7)	
*Clinical tumour stage*								
cT1	133,617 (67.7)	132,434 (68.1)	1183 (38.6)	<0.001	130,790 (68.5)	129,020 (69.1)	1770 (39.8)	<0.001
cT2	56,131 (28.4)	55,032 (28.3)	1099 (35.8)		54,503 (28.4)	52,962 (28.3)	1541 (34.7)	
≥cT3	5298 (2.7)	4680 (2.4)	618 (20.2)		5336 (2.7)	4265 (2.3)	1071 (24.1)	
Unknown	2362 (1.2)	2196 (1.1)	166 (5.4)		679 (0.4)	615 (0.3)	64 (1.4)	
*Biopsy Gleason grade group*								
1	86,012 (43.6)	85,888 (44.2)	124 (4)	<0.001	82,691 (43.2)	82,523 (44.2)	168 (3.8)	<0.001
2	53,921 (27.3)	53,688 (27.6)	233 (7.6)		52,570 (27.5)	51,951 (27.8)	619 (13.9)	
3	24,697 (12.5)	24,397 (12.6)	300 (9.8)		24,036 (12.6)	23,315 (12.5)	721 (16.2)	
4	18,780 (9.5)	18,029 (9.3)	751 (24.5)		18,293 (9.5)	17,245 (9.2)	1048 (23.6)	
5	13,998 (7.1)	12,340 (6.3)	1658 (54.1)		13,718 (7.2)	11,828 (6.3)	1890 (42.5)	

#### Validation with the use of the Briganti nomogram

Within the use of the Briganti nomogram to predict the LNM probability >10%, 117,120 assessable patients were identified (Table 2a). Here, a pelvic CT/MRI was not recommended in 91,374 (78.0%) patients. Within those individuals 493 (0.4%) with established locoregional LNM would have been missed. This resulted in a NPV of 99.5%. Conversely, a pelvic CT/MRI was recommended in 25,746 (22.0%) patients. Of those, 23,695 (20.2%) would have been imaged despite harbouring cN0 status. The resulting PPV was 8.0% and the resulting NNI to detect one patient with locoregional LNM was 12.5. When patient stratification according to NCCN pelvic CT/MRI recommendations were assessed according to sensitivity and specificity values, a sensitivity of 80.6% and a specificity of 79.3% were recorded. Finally, the positive and negative likelihood ratios were 3.89 and 0.24, respectively.

**Table 2:** Recommendations for pelvic CT/MRI according to the NCCN prostate cancer guidelines, >10% LNM probability predicted **a** with the use of the Briganti nomogram and **b** with the use of the online MSKCC nomogram

a) NCCN recommends pelvic CT/MRI if clinical tumour stage T3 or T4 or if T1 or T2 and >10% LNM probability			
Overall population * n* = 117,120	Reference: cN1 stage according to imaging		NNI = 12.5 (1/PPV)
	cN1 * n* = 2544 (2.2%)	cN0 * n* = 114,576 (97.8%)	
*Pelvic CT/MRI recommended* * n* = 25,746 (22.0%)	TP * n* = 2,051 (1.8%)	FP * n* = 23,695 (20.2%)	PPV = TP/(TP + FP) 8.0%
*Pelvic CT/MRI not recommended* * n* = 91,374 (78.0%)	FN * n* = 493 (0.4%)	TN * n* = 90,881 (77.6%)	NPV = TN/(FN + TN) 99.5%
	Sensitivity = TP/(TP + FN) 80.6%	Specificity = TN/(FP + TN) 79.3%	Accuracy = TP + TN/All 79.3%

b) NCCN recommends pelvic CT/MRI if clinical tumour stage T3 or T4 or if T1 or T2 and >10% LNM probability			
Overall population * n* = 119,502	Reference: cN1 stage according to imaging		NNI = 12.8 (1/PPV)
	cN1 * n* = 2892 (2.4%)	cN0 * n* = 116,610 (97.6%)	
*Pelvic CT/MRI recommended* * n* = 31,982 (26.8%)	TP * n* = 2497 (2.1%)	FP * n* = 29,485 (24.7%)	PPV = TP/(TP + FP) 7.8%
*Pelvic CT/MRI not recommended* * n* = 87,520 (73.2%)	FN * n* = 395 (0.3%)	TN * n* = 87,125 (72.9%)	NPV = TN/(FN + TN) 99.5%
	Sensitivity = TP/(TP + FN) 86.3%	Specificity = TN/(FP + TN) 74.7%	Accuracy = TP + TN/All 75.0%

*NCCN* National Comprehensive Cancer Network, *CT* computer tomography, *MRI* magnetic resonance imaging, *LNM* lymph node metastases, *TP* true positive, *FP* false positive, *FN* false negative, *TN* true negative, *NNI* number needed to image, *PPV* positive predictive value, *NPV* negative predictive value

#### Validation with the use of the MSKCC nomogram

Within the use of the MSKCC nomogram to predict the LNM probability >10%, 119,502 assessable patients were identified (Table 2b). Here, a pelvic CT/MRI was not recommended in 87,520 (73.2%) patients. Within those individuals 395 (0.3%) with established locoregional LNM would have been missed. This resulted in a NPV of 99.5%. Conversely, a pelvic CT/MRI was recommended in 31,982 (26.8%) patients. Of those, 29,485 (24.7%) would have been imaged despite harbouring cN0 status. The resulting PPV was 7.8% and the resulting NNI to detect one patient with locoregional LNM was 12.8. When patient stratification according to NCCN pelvic CT/MRI recommendations were assessed according to sensitivity and specificity values, a sensitivity of 86.3% and a specificity of 74.7% were recorded. Here, the positive and negative likelihood ratios were 3.41 and 0.18, respectively.

### NCCN recommendations to perform a bone scan

#### Study population

Of 197,408 assessable patients (Table 1), 1.6% (*n* = 3066) harboured bone metastases. Patients with bone metastases were significantly older (70 vs. 65 years, interquartile range: 62–77 vs. 59–71, *p* < 0.001), more frequently had a PSA value >20 ng/ml (57.5 vs. 7.4%, *p* < 0.001), more frequently harboured clinical tumour stage T3 to T4 (20.2 vs. 2.4%, *p* < 0.001) and more frequently harboured GGG 5 (54.1 vs. 6.3%, *p* < 0.001).

#### Validation

Within the 197,408 assessable patients, a bone scan was not recommended in 148,600 (75.3%) patients (Table 3). Within those individuals, 281 (0.14%) with established bone metastases would have been missed. This resulted in a NPV of 99.8%. Conversely, a bone scan was recommended in 48,808 (24.7%) patients. Of those, 46,023 (23.32%) would have been imaged despite harbouring M0 status. The resulting PPV was 5.7% and the resulting NNI to detect one patient with bone metastases was 17.5. When patient stratification according to NCCN bone scan recommendations were assessed according to sensitivity and specificity values, a sensitivity of 90.8% and a specificity of 76.3% were recorded. Finally, the positive and negative likelihood ratios were 3.83 and 0.12, respectively.

**Table 3:** Recommendations for bone scan according to the NCCN prostate cancer guidelines

NCCN recommends bone scan if PSA > 20 in clinical tumour stage T1 or if PSA > 10 ng/ml and clinical tumour stage T2 or if clinical tumour stage T3 or T4 or if Gleason score ≥ 8			
Overall population * n* = 197,408	Reference: M1b stage according to imaging		NNI = 17.5 (1/PPV)
	M1b * n* = 3066 (1.55%)	M0 * n* = 194,342 (98.45%)	
*Bone scan recommended* * n* = 48,808 (24.7%)	TP * n* = 2785 (1.41%)	FP * n* = 46,023 (23.32%)	PPV = TP/(TP + FP) 5.7%
*Bone scan not recommended* * n* = 148,600 (75.3%)	FN * n* = 281 (0.14%)	TN * n* = 148,319 (75.13%)	NPV = TN/(FN + TN) 99.8%
	Sensitivity = TP/(TP + FN) 90.8%	Specificity = TN/(FP + TN) 76.3%	Accuracy = TP + TN/All 76.5%

*NCCN* National Comprehensive Cancer Network, *PSA* prostatic specific antigen, *TP* true positive, *FP* false positive, *FN* false negative, *TN* true negative, *NNI* number needed to image, *PPV* positive predictive value, *NPV* negative predictive value

## Discussion

Staging of patients with PCa provides vital information with respect to clinical decision-making. Confirmation of locoregional LNM affects treatment choice. Similarly, confirmation of bone metastases, also affects treatment assignment. NCCN guidelines have defined criteria for imaging of patients with suspected locoregional LNM.^2^ Similarly, the NCCN guidelines have also established criteria for imaging of patients in whom bone metastases are suspected.^2^ However, to the best of our knowledge these recommendations have never been validated in a community setting within a large epidemiological database. Based on this limitation we tested the performance characteristics of these guideline recommendations. Specifically, we hypothesised that the use of the NCCN guidelines will not result in more than a marginal proportion of patients with missed locoregional lymph invasion or missed bone metastases. Our analyses demonstrated several noteworthy findings.

First, regarding the NCCN recommendations to perform a pelvic CT/MRI we tested two different approaches. Specifically, we tested the use of two different (Briganti et al. and online MSKCC) LNM nomograms to predict the LNM probability >10%, since no specification within the NCCN guidelines is made with respect to, which nomogram should be used. With the use of the Briganti nomogram, 117,120 assessable patients were identified, and a pelvic CT/MRI was not recommended in 91,374 (78.0%) patients. Of those, 493 (0.4%) did harbour locoregional LNM and would be considered as missed. Similarly, with the use of the online MSKCC nomogram, 119,502 assessable patients were identified, and a pelvic CT/MRI was not recommended in 87,520 (73.2%) patients. Of those, 395 (0.3%) did harbour locoregional LNM and would be considered as missed instances of LNM. This marginally low number of patients resulted in a virtually perfect NPV of 99.5%, for both nomograms. In consequence, we confirmed that the NCCN guideline recommendations for pelvic lymph node imaging result in only a marginal proportion of omitted instances of imaging, despite the presence of locoregional LNM, regardless of the used nomogram to predict the LNM probability >10%. Based on this observation, the use of the NCCN recommendations for identification of LNM can be safely endorsed in the community setting.

We also examined the guidelines regarding their sensitivity and specificity. Here, a sensitivity of 80.6% with the use of the Briganti et al. nomogram and respectively 86.3% with the use of the MSKCC nomogram was recorded in individuals with established locoregional LNM. Conversely, the specificity with the use of the Briganti et al. nomogram was 79.3% compared to 74.7% with the use of the MSKCC nomogram, in individuals without locoregional LNM. Higher sensitivity of the MSKCC nomogram could be related to the fact that the Briganti nomogram was developed within a European tertiary referral center.^5^ Conversely, the MSKCC nomogram originates from an American tertiary care center.^6^ Despite these small differences, the performance characteristics recorded for both nomograms indicate an excellent balance between sensitivity and specificity that is expected from a robust testing recommendation. Moreover, the use of both nomograms resulted in similar NNI values, which were, respectively, 12.5 for the Briganti nomogram vs. 12.8 for the MSKCC nomogram. Finally, the positive and negative likelihood ratios with the use of the Briganti vs. the MSKCC nomogram were similar, 3.89 and 0.24 vs. 3.41 and 0.18, respectively. These results demonstrate an acceptable confidence for both nomograms regarding the NCCN recommendations to perform a pelvic CT/MRI. Taken together, our data validate the use of the NCCN guideline recommendations for identification of LNM with either the MSKCCC or the Briganti nomogram. The choice of a specific nomogram may be left to the discretion of individual physicians.

Second, regarding the NCCN guideline recommendations for presence of bone metastases, we relied on 197,408 assessable patients. Within those individuals the guidelines did not recommend a bone scan in 148,600 (75.3%) patients. Of those, only 281 or 0.14% did harbour bone metastases and would be considered as missed instances of bone metastases. This marginally low number and proportion of patients resulted in a virtually perfect NPV of 99.8%. In consequence, we confirmed that the NCCN guideline recommendations for bone metastases imaging are safe and result in a marginal proportion of omitted instances of imaging, despite the presence of bone metastases.

We also examined the guideline recommendations for bone imaging regarding their sensitivity and specificity. Here, a sensitivity of 90.8% was recorded in individuals with established bone metastases. Conversely, a specificity of 76.3% was recorded in individuals without bone metastases. These values indicate an excellent balance between sensitivity and specificity that can be expected from a well-balanced testing recommendation. The sensitivity in our analyses was noticeably higher than the one reported previously by Merdan et al. (82.3%), who tested the bone scan recommendations from the NCCN guidelines within the Michigan Urological Surgery Improvement Collaborative (MUSIC) clinical registry.^7^ However, Merdan et al. relied on a smaller cohort (*n* = 1509) compared to the current study (*n* = 197,408). The recorded sensitivity in the current study was also higher than the one reported by Briganti et al.^8^ (79.2%), who relied on an even smaller (*n* = 853) and more historical cohort (2003–2008) from a single European tertiary referral center. Taken together, the bone scan recommendations from the NCCN guidelines demonstrate a high sensitivity in our large patient cohort, which results in a low proportion of missed patients that harbour bone metastases. However, the NNI of 17.5 demonstrates that the staging recommendations from the NCCN guidelines are not perfect in distinguishing between patients with or without bone metastases. Finally, the positive- and negative-likelihood ratios of 3.83 and 0.12, respectively, demonstrate acceptable confidence for clinicians to rely on the NCCN guideline recommendations when to perform bone imaging.

Taken together, our analyses demonstrated that the NCCN guideline staging recommendations for prediction of LNM, as well as those for prediction of bone metastases result only in a marginal number of missed patients with either locoregional LNM or bone metastases in a large North American community setting, such as the SEER database. This is an important information, since it demonstrates that with the use of these recommendations most patients with metastatic disease can be identified. To the best of our knowledge, this is the first report of this kind and further studies are welcome to confirm our results.

Our study is not devoid of limitations. First and foremost, the SEER database only represents approximately 30% of the United States population, which is a limitation for itself.^9^ Moreover, the NCCN guidelines do not provide recommendations for abdominal CT/MRI staging and thereby we were not able to include patients with visceral metastases or non-locoregional LNM in our analyses. However, such patients need also be identified and deserve to be studied, since their outcome and treatment differs from those of patients with localised PCa. Additionally, the SEER database does not capture information in regards of symptoms (e.g., bony pains) or laboratory abnormalities (e.g., elevated alkaline phosphatase) and as such, the current study cannot account for cases that might be diagnosed based on this information. Last but not least, both recommendations are based on gold standards that maybe suboptimal.^10,11^ Specifically, bone scans have recently been shown to miss bone metastases.^12,13^ Similarly, pelvic CT/MRI have recently been shown to miss locoregional LNM.^14–16^ In consequence, future gold standards will need to be reassessed. However, to date only studies with very limited sample sizes are available, when new gold standards such as prostate specific membrane antigen scans are compared to routine imaging.^15^ In consequence, population-based studies relying on prostate specific membrane antigen-based testing cannot yet be performed. Limited availability of prostate specific membrane antigen testing will persist for next several years and will hamper such efforts for at least next decades.

## Conclusion

The NCCN based recommendation for locoregional LNM miss very few patients with clinical LNM (0.3–0.4%), regardless of the applied methodology and provide a virtually perfect NPV of 99.5% and acceptable negative likelihood ratios (0.18–0.24). The NCCN bone scan recommendations also miss a marginal number of patients with established bone metastases (0.14%) and yield a virtually perfect NPV of 99.8% and a very acceptable negative likelihood ratio of 0.12. In consequence, both NCCN recommendations may be safely endorsed in clinical practice.

## Availability of data and materials

SEER data is available for purchase at https://seer.cancer.gov.
